# Representational geometry: integrating cognition, computation, and the brain

**DOI:** 10.1016/j.tics.2013.06.007

**Published:** 2013-08

**Authors:** Nikolaus Kriegeskorte, Rogier A. Kievit

**Affiliations:** 1 Medical Research Council, Cognition and Brain Sciences Unit, Cambridge, UK; 2 Department of Psychological Methods, University of Amsterdam, Amsterdam, The Netherlands

## Abstract

The cognitive concept of representation plays a key role in theories of brain information processing. However, linking neuronal activity to representational content and cognitive theory remains challenging. Recent studies have characterized the representational geometry of neural population codes by means of representational distance matrices, enabling researchers to compare representations across stages of processing and to test cognitive and computational theories. Representational geometry provides a useful intermediate level of description, capturing both the information represented in a neuronal population code and the format in which it is represented. We review recent insights gained with this approach in perception, memory, cognition, and action. Analyses of representational geometry can compare representations between models and the brain, and promise to explain brain computation as transformation of representational similarity structure.

## Highlights

• Representational geometry is a framework that enables us to relate brain, computation, and cognition.
• Representations in brains and models can be characterized by representational distance matrices.
• Distance matrices can be readily compared to test computational models.
• We review recent insights into perception, cognition, memory, and action and discuss current challenges.

## The representational geometry of neuronal population codes

The concept of representation is central to the cognitive and brain sciences. We interpret neuronal activity as serving the function of representing content, and of transforming representations of content, with the ultimate objective to produce successful behaviors. The content could be a visual image, a sound or odor, a semantic interpretation of sensory input, a proposition, a goal, a planned action, or a motor sequence. The representational interpretation [1] provides a powerful explanatory framework that makes it easier to understand neuronal activity in the context of the overall function of the brain. Representation links cognition to brain activity and enables us to build functional theories of brain information processing [2].

Neurophysiology has long interpreted the selectivity of neurons as serving to represent various kinds of sensory and higher-level information. The population of neurons within an area is thought to jointly represent the content in what is called a neuronal population code [3]. It is the pattern of activity across neurons that represents the content. The many possible combinations of activity states of neurons provide a rich representational space. Motivated by this idea, recent analyses of neuronal recordings and functional imaging data have increasingly focused on patterns of activity across many neurons within a functional region [4].

We can think of a brain region's representation as a multidimensional space. The dimensions of the space correspond to the neurons, and a point corresponds to an activity pattern (i.e., each neuron's activity provides the coordinate value for one of the dimensions). A visually perceived object, for example, will correspond to a point in the representational space of a given visual area. The set of all possible objects (or pieces of mental content) corresponds to a vast set of points in the space. It is the geometry of these points that defines the nature of the representation.

**Figure 1:**
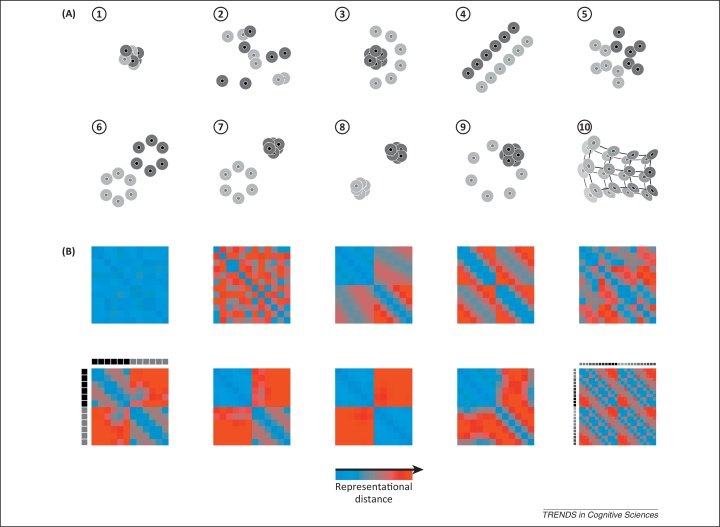
Representational geometries and their reflection in distance matrices. **(A)** Illustration of ten hypothetical representational geometries for brain regions (numbered 1–10). Each dot corresponds to the representation of a particular piece of content (e.g., a visual object). The space in which the dots are placed is the space of representational patterns (illustrated as two-dimensional, but high-dimensional in reality). The halo regions around the dots indicate the margin of error; dots with overlapping error halos are indistinguishable. The items fall into two categories (dark or light), or in the case of geometry 10, on a continuous manifold (shades of gray). (1) No item is distinct from any other item. (2) Most items are distinctly represented but the categories cannot be separated by any simple boundary. (3) Only the light items are distinctly represented and they are separable from the dark items by a quadratic boundary. (4) Dark and light items are linearly separable and arranged along parallel lines with pairs of dark and light dots matched up across the boundary. (5) The items form a single cluster but the categories are linearly separable. (6) The items form two category clusters that are linearly separable and within which all items are distinct. (7) Like the previous case, but the items in the dark category are indistinguishable. (8) Like the previous case, but only the category distinction is represented; items within each category are indistinguishable from each other. (9) The dark items are indistinguishable and located among the distinctly represented light items on a circle. (10) Items fall on two manifolds that closely follow each other, with pairs of items matched up across them. **(B)** Representational distance matrix for each of the ten geometries (in the corresponding panel location). Distances are color-coded from blue (items indistinguishable) to red (items widely separated). Each matrix is indexed vertically (from the top down) and horizontally (from left to right) by the items as illustrated in the lower left panel. Only geometry 10 (lower right) has a different item set, and the upper left quadrant corresponds to the front manifold and the lower right quadrant to the back manifold. See Box 2 for actual brain representations exhibiting some of the geometric features illustrated here.

Mathematical and cognitive psychology have a long history of investigations of representational geometry on the basis of behavioral data 5, 6, 7, 8, 9, 10. However, the notion of representational geometry has only more recently been brought into the analysis of brain-activity data 11, 12, 13, 14, 15. To characterize the geometry of a representation, we can compare the brain-activity patterns representing a set of stimuli (or, more generally, experimental conditions) to each other. The dissimilarity of two patterns corresponds to the distance between their points in the representational space. Having measured these distances, we can construct a matrix, the representational dissimilarity matrix (RDM), in which we can look up the representational distance (or dissimilarity) for each pair of stimuli (Figure 1). Intuitively, the RDM tells us which distinctions between stimuli the population code honors and which distinctions it disregards.

Considering RDMs makes it very easy to compare different representations (e.g., different brain regions, a region to a computational model representation, or the same region between different individuals or species) by just computing the correlation between the RDMs (Box 1). Comparing activity patterns directly, by contrast, would require us to define the correspondence mapping between, say, voxels of two regions, or between single neurons and the units of a computational network model, or between voxels of the same region in two individuals. Establishing these mappings can be difficult and generally requires a separate experimental data set 16, 17, 18, 19. The ‘representational dissimilarity trick’ obviates the need for such correspondence mappings.

Box 1 **Representational similarity analysis**

Representational similarity analysis (RSA) is pattern information analysis that compares representational geometries between brain regions, stimulus descriptions, conceptual and computational models, and behavioral reflections of similarity [15]. It can be applied to functional imaging data (including fMRI, MEG, and EEG) as well as neuronal recording data. The three basic steps (Figure I) are as follows. (i) Choose a brain region and estimate the activity patterns. The region can be functionally or anatomically defined. The patterns can be estimated with standard methods used in univariate analyses. In fMRI, for example, a linear model with a hemodynamic response predictor for each stimulus might be used to estimate the response of each voxel to each stimulus. For neuronal recordings, a windowed spike count might be used. However, any other features of the responses, such as features reflecting the temporal response structure or energy in different frequency bands, could equally be used to define the representation of each stimulus. (ii) Estimate the representational dissimilarity matrix (RDM). The representation in a given brain region or computational model is characterized by the matrix of dissimilarities between the stimulus representations. A popular distance measure is the correlation distance (1–Pearson correlation across voxels, neurons, features, or model units). The correlation distance disregards the overall activation level (spatial mean), rendering the analysis complementary to analyses of overall activation. Other distance measures such as the Euclidean or Mahalanobis distance, or cross-validated measures such as the discriminant *t* value or accuracy can also be used. (iii) Compare RDMs from brains, behaviors, and models. The key step is to compare RDMs to assess to what extent different representations are alike. We might want to know whether a brain representation (a) reflects stimulus properties, (b) reflects higher-level semantic properties, (c) can be accounted for by a computational model, (d) reflects representations in other brain regions, (e) is similar to a putative homologous representation in another species, or (f) is reflected in behavior, for example in similarity judgments, in stimulus confusions, or in reaction times in discrimination tasks. One useful way to compare RDMs is to compute the correlation between the corresponding dissimilarities. The rank correlation (Spearman) is often used for this purpose when a linear relationship between the dissimilarities cannot be assumed (e.g., when comparing fMRI-based RDMs to other RDMs). Statistical inference is commonly performed by means of randomization testing (randomly permuting the stimulus labels to simulate the null distribution of the RDM correlation) and bootstrap techniques (to compare the relative performance of different models). Comparing two representations at the level of dissimilarities rather than at the level of the original patterns is a useful trick that obviates the need for defining the correspondence mapping between the representational units. Like classifier decoding, RSA is a pattern information technique that is sensitive to information encoded combinatorially in fine-grained patterns of activity. However, rather than asking what information can be (linearly) read from the representation, RSA attempts to characterize the representational geometry and compare it to various models. Like encoding models (also known as voxel or population receptive field models in the fMRI literature 17, 18, 112), RSA captures the representation of a rich set of stimuli and aims to test computational models of brain information processing that generalize to novel stimuli. However, rather than comparing brains and models at the level of activity patterns (requiring fitting of weights that define the relationship between model units and voxels), RSA compares representations at the level of dissimilarity matrices.

**Figure I:**
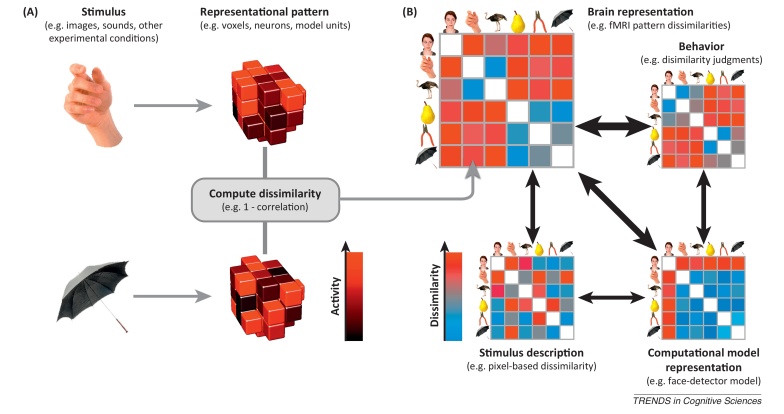
Representational similarity analysis. Illustration of the steps of RSA for a simple design with six visual stimuli. **(A)** Stimuli (or, more generally, experimental conditions) are assumed to elicit brain representations of individual pieces of content (e.g., visual objects). Here the representation of each item is visualized as a set of voxels (an fMRI region of interest) that are active to different degrees (black-to-red color scale). We compute the dissimilarity for each pair of stimuli, for example using 1–correlation across voxels. **(B)** The representational dissimilarity matrix (RDM) assembles the dissimilarities for all pairs of stimuli (blue-to-red color scale for small-to-large dissimilarities). The matrix can be used like a table to look up the dissimilarity between any two stimuli. The RDM is typically symmetric about a diagonal of zeros (white entries along the diagonal). RDMs can similarly be computed from stimulus descriptions (bottom left), from internal representations in computational models (bottom right), and from behavior (top right). By correlating RDMs (black double arrows), we can then assess to what extent the brain representation reflects stimulus properties, can be accounted for by different computational models, and is reflected in behavior.

A popular method for testing whether two classes of stimuli can be discriminated in the representation is pattern classifier analysis 20, 21, 22, 23, 24, 25, 26, 27. Classifier analysis, or decoding, typically focuses on binary distinctions and on revealing whether a region contains information about the class of a stimulus. In practice, the classifier is often linear and successful classification indicates some degree of linear discriminability between the classes. The analysis of representational geometry is complementary to classifier analysis and goes beyond the question of discriminability of classes (and the presence of information). Two classes of stimuli can be discriminable for many different representational geometries (Figure 1, scenarios 2–9). However, the particular geometry matters for the computational function of the region. Beyond the question of what information is present (i.e., pieces of content distinctly represented) and what information is explicit (e.g., in the sense of being amenable to linear readout), a representation imposes a rich structure on the domain of its content. The detailed geometry of the representations of particular items can reflect their similarity, their categorical divisions, and their continuous variation along property dimensions of behavioral significance. Items that are clustered in a representation can easily be grouped together, and their differences abstracted from, when the code is read by other brain regions. Representational geometry thus provides a basis for generalization and abstraction, important hallmarks of cognition [28]. In fact, brain computation can be construed as the transformation of the representational geometry along stages of processing [29].

Recent papers have reviewed results from pattern decoding of visual representations [27] and pattern-information methods for testing computational models [30]. Here we give an overview of some of the insights from recent studies of representational geometry. The next section covers vision, the field that has been most active with this approach. We then describe applications beyond vision, addressing other sensory modalities, memory, cognition, and action from this perspective. Finally we highlight current challenges and future directions for studies of representational geometry.

## Representational geometry in the visual system

The most rigorous account of a sensory representation is provided by a computational model that predicts neuronal responses to arbitrary stimuli. If we can accurately predict the responses of all neurons in an area, we have captured the computations up to that area. This method has been very successful for V1 and is being extended to higher-level cortical representations. If we could predict neuronal responses throughout the brain, along with behavioral output, we might not need the abstraction of representational geometry, or indeed the concept of representation. However, even for V1, the degree to which we can predict responses is limited [31]. Predicting neural responses becomes more difficult as we move to higher-level regions. The space of computational mechanisms and model parameters becomes very complex, making it hard to consider all plausible models and to adjudicate between them with the limited amounts of data we can acquire. It is useful therefore to first seek a more abstract descriptive characterization of the population code for each area. Analyses of representational geometry have brought insights into all stages of visual representation, from early visual areas to high-level object representations in the ventral stream.

**Figure 2:**
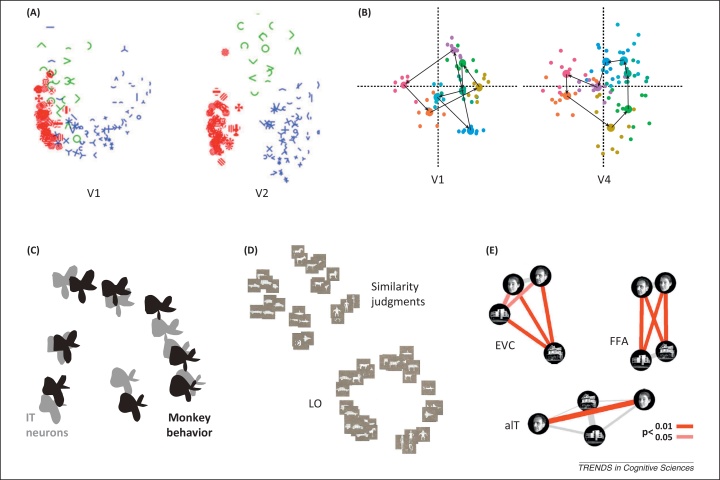
Representational geometries visualized by arranging the stimuli in two dimensions. An intuitive way of jointly visualizing the many pairwise high-dimensional distances that characterize a representation is to arrange the stimuli in two dimensions. This figure shows the stimuli presented in four studies (panels A–D) arranged in 2D such that stimuli eliciting similar response patterns are placed together and stimuli eliciting different response patterns are placed apart. **(A)** Hegdé and Van Essen investigated early visual neuronal population representations of grayscale symbols and patterns [32]. The stimuli are colored here according to the three clusters they formed in V2. Reproduced, with permission, from [32]. **(B)** Brouwer and Heeger found that the representation of colors (as shown) reflects perceptual color space (connection lines) in V4, but not in V1, despite high within-color clustering in V1, indicating color decodability [38]. Analyses were based on fMRI response patterns. Reproduced, with permission, from [38]. **(C)** Op de Beeck *et al.* studied the representation of parameterized shapes in monkey inferior temporal (IT) neurons and its reflection in the animals’ behavioral judgments [13]. Shape parameters were smoothly reflected in both the IT representation (gray) and behavioral judgments (black), whose independently performed multidimensional scaling (MDS) arrangements are superimposed here for comparison. Reproduced, with permission, from [13]. **(D)** Edelman *et al.* investigated the representation of shaded renderings of 3D models of animals and vehicles in human visual cortex with fMRI [11]. Similarity judgments and fMRI activity patterns in lateral occipital (LO) cortex reflected categorical divisions. Reproduced, with permission, from [11]. **(E)** Kriegeskorte *et al*. examined the representation of face and house images along stages of the ventral stream 15, 63. The fMRI patterns from early visual cortex (EVC) significantly discriminated (red lines) all physically dissimilar images; the fusiform face area discriminated the two categories; and an anterior IT (aIT) face-identity region discriminated the physically similar individual faces. Reproduced, with permission, from 15, 63. The arrangements in all panels were computed from the response pattern dissimilarities by MDS, except for (B), where the space spanned by the first two principal components is shown.

For example, Hegdé and Van Essen found that considering the population representational geometry revealed clearer distinctions between early visual areas than single-cell selectivity measures [32]. Neuronal population response patterns elicited by symbols and visual patterns showed clustering according to complexity categories in V2 and V4, but not in V1 (Figure 2A). Freeman *et al.* found a clear distinction between V1 and V2 when investigating the representation of natural textures [33]. V2 neuronal representational distances better reflected perceived texture similarities [34]. Human functional magnetic resonance imaging (fMRI) has suggested that the perceived similarity of natural textures, including metal, wood, glass, and fur, is best reflected in the representational geometry of higher ventral-stream regions (Box 2, Figure IB) [35]. Whereas V1 may represent local Fourier statistics, V2 and higher regions might compute higher-order statistics of V1 outputs, which are more predictive of the perceptual quality of a texture (Box 2, Figure IG) 36, 37.

Box 2 **Representational geometries in recent studies**

Inspection of the representational dissimilarity matrices (RDMs) that characterize brain regions, various models, and behavioral data is an important exploratory process that can reveal interesting and unexpected representational structure. Here we consider RDMs from a wide range of recent studies. Note that prior hypotheses and statistical inference, as reported in the original papers, are required to support any theoretical conclusions. **(A)** RDMs for human IT and explicit behavioral dissimilarity judgments reveal related yet distinct structures. Both IT and judgments emphasize animate/inanimate and face/body divisions. However, the judgments additionally emphasize human/nonhuman and man-made/natural divisions [54]. Note that the categorical structure is obvious only because the stimuli are ordered by category. In contrast to 2D arrangements by representational dissimilarity (see Figure 2 in main text), which do not depend on any choice of stimulus order, the appearance of an RDM depends on the order chosen for display. **(B)** The early visual representation of natural textures, including metal, ceramic, and glass, resembles a model based on low-level image features. The representation in the fusiform gyrus is distinct from the early visual representation and more consistent with human perceptual similarity ratings [35]. **(C)** RDMs from neuronal recordings in macaque middle and anterior face patches illustrate the transformation of representational geometry across stages of processing. Neuronal population response patterns cluster by face view in the middle face patches (left RDM). The blocks of similar patterns correspond to faces of different identities in the same view. By contrast, the anterior face patch (right RDM) exhibits strong view tolerance and selectivity for individual identities. Each identity elicited similar response patterns when presented in different views (subtle dark diagonal lines) [67]. **(D)** Images of animals from six biological species are represented distinctly in early visual and lateral occipital cortex. The early visual RDM resembles that from a computational model of V1. The lateral occipital RDM resembles judgments of biological similarity, which reflect the categories (insects, birds, primates) [53]. **(E)** An RDM model of gaze direction is contrasted with two competing models: a low-level model of physical stimulus features and a categorical model of gaze direction relative to the observer. Searchlight representational similarity analysis (not shown) revealed that anterior STS might represent gaze direction with tolerance to head view [80]. **(F)** RDM of whole-brain activity patterns during pain perception and other mental states. The RDM reveals the similarity in terms of global brain activity of eight mental states, suggesting shared recruitment of specialized brain regions in pain, emotion, interoception, and reward [113]. Yarkoni *et al.* have presented a comprehensive meta-analytical framework for analyzing the discriminability of mental states from whole-brain activity patterns [114]. Note that the representational interpretation appears less natural for whole-brain patterns reflecting various kinds of task processing than for the localized representations in the other studies discussed here. However, similar caveats apply at both levels of analysis. **(G)** The RDM for a set of natural images (based on spatial human EEG patterns 101 ms after stimulus onset) is substantially correlated with an RDM predicted by a model of spatially pooled image-contrast statistics, namely the parameters of a Weibull fit to the distribution of local contrast measurements [37].

**Figure I:**
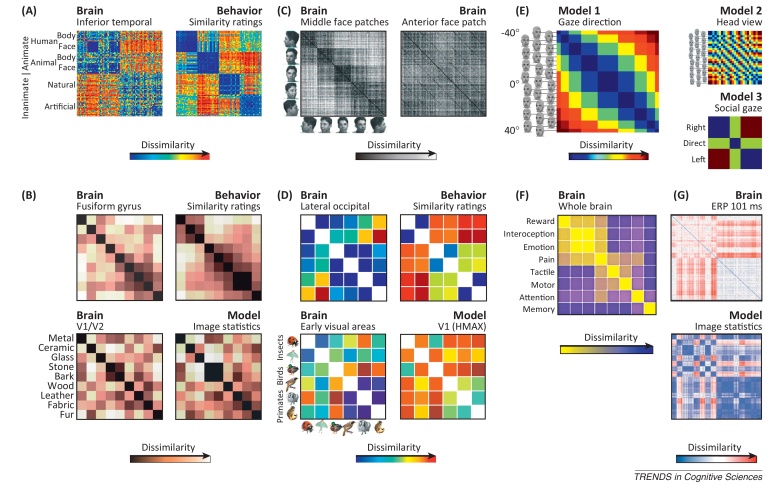
Representational dissimilarity matrices in recent studies. **(A)** RDM from fMRI patterns in human IT and RDM based on human dissimilarity judgments. Reproduced, with permission, from [54]. **(B)** RDMs from fMRI responses to natural visual textures from a model of low-level image statistics and from human similarity ratings. Reproduced, with permission, from [35]. **(C)** RDMs from neuronal recordings for monkey middle face patches (middle lateral and middle fundus, 121 neurons) and anterior face patch (anterior medial, 158 neurons). The stimuli are faces of different identities and views. One photograph per view labels a set of rows and columns for different identities in that view. Reproduced, with permission, from [67]. **(D)** RDMs from fMRI responses to images of animals from six species in three biological categories and from a computational model of V1 and subject judgments of biological similarity. Reproduced, with permission, from [53]. **(E)** RDMs for three different models of representation of faces and eye positions: model 1, by gaze direction; model 2, by head view (ignoring eyes); and model 3, by gaze categories relative to the observer (direct/eye contact, left, right). Reproduced, with permission, from [80]. **(F)** Whole-brain activity pattern dissimilarities between different functional states. Global patterns were estimated meta-analytically. Reproduced, with permission, from [113]. **(G)** RDMs based on spatial patterns of human event-related-potential amplitudes evoked by natural Images 101 ms after stimulus onset (top) and a model based on parameters of a Weibull fit to the spatially pooled distribution of local contrast measurements (bottom). Reproduced, with permission, from [37].

The transformation from a low-level feature representation to a representation that reflects perceptual qualities is a common theme among studies of representational geometry. In the domain of color, a human fMRI study [38] showed that the representational geometry in V4, but not V1–3, reflects perceptual color space, although color decoding was most accurate in V1 (Figure 2B). This illustrates the need to reveal not only what information is present in a region but also its representational geometry to understand the neural basis of perception.

Whereas textures and colors make the ‘stuff’ of vision, a major function of the ventral stream is the visual recognition of ‘things’ [39]. Ventral-stream representational geometry has been investigated using abstract parameterized shapes, which serve as a stepping stone towards real-world object images. Op de Beeck *et al.* found that the parameters of a simple 2D shape space were reflected in the representational geometry in monkey inferior temporal (IT) and in perceptual judgments of monkeys and humans (Figure 2C) [13]. Human fMRI studies similarly support a representation of shape reflecting perception in lateral occipital complex (LOC) [40], with the anterior LOC reflecting perceptual similarities most strongly [41] and lateral LOC tuned to smaller features [42]. More complex 3D shape parameterizations have also been successfully used to model IT single-neuron responses [43], suggesting that IT represents 3D shape.

Parameterized shapes afford good experimental control, but they lack naturalism and behavioral relevance. This has motivated the use of real-world photos depicting faces, people, animals, objects, and scenes. Several studies using photos suggest that ventral-stream regions do not merely represent objects in a continuous space of high-level visual features, but emphasize categorical boundaries and semantic dimensions of the visual images in humans 11, 20, 44, 45 (Figure 2D) and monkeys [46]. Both the categorical divisions and the within-category representational geometry are strikingly similar between monkey IT and human ventral–temporal object-sensitive cortex [45]. Beyond the presence of category information [20], several studies suggest that response patterns elicited by images of the same category form clusters in ventral–temporal response-pattern space 11, 45, 46. The major categorical divisions are between animates and inanimates 45, 46, 47, 48 and between faces and bodies. Such clustering was not observed in early visual representations or computational visual features of a range of complexities [45], suggesting that the clusters do not simply reflect visual feature similarity. Instead, the ventral temporal code might be optimized to emphasize behaviorally relevant categorical divisions and semantic dimensions 44, 45, 46, 47, 48, 49. The geometric centrality of an object in the representation has been linked to the perception of typicality [49]. Animates appear to form a representational cluster not only in IT but also in the amygdala [50]. The representation appears to be sensitive to the dimension of animacy, even when comparing real faces and physically similar mannequin faces [51], strengthening the case for a semantic component to the code. The animate–inanimate division is associated with a large-scale lateral-to-medial gradient in ventral temporal cortex, which is unaltered in congenitally blind individuals [52], suggesting that it does not require visual experience to develop. Finer categorical divisions within animates have also been observed in monkeys [46] and humans (Box 2, Figure ID) [53].

Human ventral–temporal representational distances closely match human dissimilarity judgments 11, 40, 41, 54, which exhibit the same major categorical divisions and a similar within-category structure. However, human judgments transcend the ventral–temporal representation in that they additionally emphasize the division between human and non-human animals and that between man-made and natural objects, which are not very pronounced in the ventral stream (Box 2, Figure IA) [54].

Places [55] and faces [56] are thought to have special behavioral relevance and specialized cortical regions dedicated to their analysis. Several studies have attempted to characterize the representational geometry of these regions. Walther *et al.* found that natural scenes presented as either photographs or line drawings could be decoded from V1, the parahippocampal place area (PPA), retrosplenial cortex (RSC), and LOC 57, 58, 59. However, only in the higher visual regions did pattern dissimilarities predict behavioral confusions. Kravitz *et al.* compared the representational geometry of scenes in early visual cortex and PPA and found that early visual representation strongly reflected the distance of the perceived scene (near vs far), whereas PPA represented whether scenes were closed or open [60]. A comparison of the representational geometries of LOC, RSC, and PPA suggested that LOC represents the objects present in the scene and RSC the global spatial layout, and PPA combines information about both of these components of scenes [61]. Morgan *et al.* found that geographic distances between landmarks of a college campus were reflected in hippocampal responses when subjects familiar with the location of the landmarks viewed them in a scanner [62]. The hippocampus responded more strongly to changes spanning a greater physical distance. However, response-pattern dissimilarity was not significantly correlated with physical distance anywhere in the brain.

Studies of face-specific representations in humans have suggested that the fusiform face area (FFA) [56] emphasizes the distinction between faces and non-faces, whereas anterior temporal cortex discriminates individual faces (Figure 2E) 63, 64, 65. However, individual-level face information has also been observed in the FFA and other regions 64, 65, 66. The best demonstration of the transformation of face representational geometry across face regions comes from fMRI-targeted neuronal recordings in monkeys (Box 2, Figure IC) [67]. The representation of identities was view-specific in the middle face patches (ML and MF), partially view-tolerant in anterior face patch (AL), with mirror-symmetric views co-localized in the representational space, and almost view-invariant in the most anterior face patch (AM). A mirror-symmetric representation of face views has also been observed in humans [68]. Many higher visual areas, including LOC, occipital face area (OFA), FFA, PPA, and dorsal regions, showed a mirror-symmetric response to faces. For example, views of –60° and 60° elicited similar response patterns, suggesting pooling of mirror-symmetric lower-level features as a computational step towards greater tolerance to view changes.

Achieving tolerance to stimulus variations that are not relevant to a given task such as face identification is one of the central challenges of object recognition [69]. Although ventral-stream representations are not fully invariant [70] to the view, position, or scale of an object, they support linear readout with some robustness to these accidental properties [71]. To understand how tolerance is achieved, Rust and DiCarlo compared the population representational geometries of V4 and IT on the basis of neuronal recordings in monkeys [72]. Both regions discriminated individual images and their scrambled counterparts. IT exhibited reduced information about scrambled images, but increased generalization for intact images across position and context clutter. This suggests a transformation of representational geometry in which position and clutter are de-emphasized relative to the presence of complex feature conjunctions diagnostic of differences between real-world objects.

A special case of clutter is the presence of additional objects in the image. Several studies have investigated ventral temporal response patterns to multiple simultaneously presented objects in monkeys [73] and humans 74, 75, 76. Results suggest that the response pattern elicited by multiple objects is well predicted by the average of the response patterns [73]. If one of the objects is attended, this object receives a greater weight in the average [75]. These results are broadly consistent with biased competition and with the divisive normalization model [77], in which the summed neural population response to multiple inputs is held constant through recurrent suppression.

As we observe people in the real world, we do not just recognize them. We infer a host of socially important pieces of information. Facial expressions of emotion appear to be represented in posterior STS with the representational geometry reflected in similarity judgments of the expressions [78]. It has been suggested that the STS and medial prefrontal regions represent perceived emotions with invariance to the sensory source (dynamic faces, bodies, or voices, [79]). Another socially important feature of faces is gaze direction. Displacement of the dark iris and pupil provides a subtle but socially important indication of where someone is looking. Carlin *et al.* reported a representation of gaze direction in right anterior STS that was tolerant to changes in both head view and physical image features (Box 2, Figure IE) [80].

Visual representations are not merely perceptual but are also involved in mental imagery in the absence of visual input. A number of human fMRI studies have investigated brain representations during visual imagery and their relationship to perceptual representation. Results support the idea that imagery and perception of the same visual content might be represented in the same cortical regions and in a similar representational format 81, 82, 83.

Overall, studies of the geometry of visual representations have impressively documented the stage-wise transformation of the retinal image from low-level representations of local physical features to high-level holistic representations of objects, faces, and scenes that better reflect perceptual qualities, emphasizing behaviorally important categories and semantic dimensions and deemphasizing accidental and behaviorally irrelevant variation of the visual input. Further studies are needed to reveal the full computational mechanism giving rise to these transformations.

## Representational geometry beyond vision

### Auditory perception

Like vision, audition requires substantial tolerance to accidental variations of the signals to be recognized. The imposition of categorical boundaries on a fundamentally continuous space of stimuli is another shared feature between the two modalities. Recent studies have investigated the representation of sound categories 84, 85. Giordano *et al.* investigated the representation of natural environmental sounds and reported that representational geometry in the planum temporale emphasized particular categorical distinctions more strongly than predicted by low-level feature models [84]. The categories were living/non-living and human/non-human, which are highly behaviorally relevant divisions similar to those emphasized in ventral visual representations.

The clearest examples of categorical representation might be expected in the domain of human language. Speech percepts are categorical not only at the level of conceptual and semantic content but also at the phonetic level. A well-known example is the categorical perception of phonemes. A recent study used human intracranial electrode arrays to investigate the representational geometry of a continuum of artificial speech sounds ranging from ‘ba’ through ‘da’ to ‘ga’ [86]. Response patterns in posterior superior temporal gyrus formed clear clusters corresponding to phonemes, despite the fact that the sound continuum was sampled in acoustically equal steps. Pattern dissimilarity emerged rapidly over time, peaking at the same time (∼110 ms) as the evoked potentials. A human fMRI study investigated representations of the phonemes /la/ and /ra/ in native speakers of English and Japanese [87]. The representational dissimilarity between /la/ and /ra/ phonemes in the right primary auditory cortex, but not the overall activation, predicted the extent to which speakers were able to discriminate between the two phonemes between, and even within the two groups.

Beyond vision and audition, a close link between representational geometry and perception has also been observed for olfactory stimuli. Behavioral similarity ratings for smells correlated with neural pattern representational similarities in the posterior piriform cortex [88]. Across several sensory modalities, studies of representational geometry have demonstrated that brain representations emphasize behaviorally relevant categorical distinctions and predict perceptual similarities.

### Memory

Recent studies have begun to investigate representations of particular items during memory encoding and retrieval, and how the precision of representational reinstatement during encoding and retrieval predicts the success or failure of these memory operations. Polyn *et al.* showed that the category-specific patterns arising during encoding of faces, locations, and objects are reinstated in a subsequent free-recall phase [89]. Activity patterns during free recall predicted the category about to be recalled several seconds in advance.

A more recent study by Xue *et al.* investigated the encoding process and found that more precise perceptual reinstatement of representations during encoding predicted better memory [90]. Interestingly, the precision of perceptual reinstatement of representations has also been associated with conscious representation [91]. Subsequent studies suggested some refinements to this picture, in which activity patterns in the hippocampus and other medial temporal regions have distinct signatures that predict successful encoding [92] and retrieval 93, 94.

In the studies described so far, memory served as a storage facility, but the structure of the representational categories was never manipulated. A recent study associated particular exemplars from each category with a shock [95]. This led to emergence in the frontoparietal representation of a new categorical division between the shock-associated and other images. Moreover, the patterns during fear learning predicted the long-term behavioral expression of fear memories [96].

In sum, studies of representational geometry are beginning to reveal how the perceptual representation of an individual piece of content affects its mnemonic encoding and how reinstatement of the representation during recall enables successful recollection. We are also beginning to elucidate the specific roles of the hippocampus and other medial temporal regions and the plasticity of the representational space itself, including the formation of new behaviorally relevant categorical divisions.

### Action and motor control

We should be able to characterize representations along the entire perception–action cycle by their representational geometry. The primary motor representation is a classic example of a population code [97]. We focus here on two recent studies that explored the geometry of motor representations. Wiestler *et al.* studied the representation of finger movements and sensations in the cerebellum [98], a structure thought to relate sensory and motor representations for smooth sensorimotor control. They compared cerebellar regions to cortical areas M1 and S1. Cerebellar and cortical representations both discriminated movements of different individual fingers. Both also discriminated sensations in different individual fingers. For a given finger, movement and sensation were associated with similar patterns in M1 and S1. In the cerebellum, however, the representations were not consistent between movement and sensation, with motor and sensory finger representations apparently randomly interdigitated. This arrangement may enable the cerebellum to associate movements and their sensory consequences in flexible ways, a requirement in learning new motor tasks. In another study, Diedrichsen *et al.* investigated co-localized motor representations of our two hands, which might serve to coordinate the hands during bimanual tasks [99]. Representations of unimanual finger movements were represented mostly in contralateral M1 and S1, with a faint echo of a symmetric representation in ipsilateral areas. Such an arrangement might facilitate symmetric bimanual movements. In premotor and parietal areas, unimanual movements also had an ipsilateral representation. However, it was not a symmetric echo, but qualitatively different. Such a co-localized representation of both hands might serve to associate the movements of the two hands in flexible ways to coordinate the hands during asymmetric bimanual tasks.

## Current challenges for investigations of representational geometry

### Testing many models: a simultaneously hypothesis- and data-driven approach

Many studies have focused on one or two models of representation. In the visual domain, this could be a particular computational model, such as a Gabor filter model, a parameterized shape model, a semantic model, or a behavioral characterization of the representational geometry. The field is in an early phase in which finding that a model explains significant variance (of neuronal or voxel responses or response pattern dissimilarities) is considered an advance. This is a low bar. The theoretical advance is not always substantial, because a great number of qualitatively different models may capture some component of the representation. For theoretical progress, we need statistical model comparisons along with estimates of the amount of non-noise variance left unexplained by each model. Ideally, we would like to cover the entire space of models that have not yet been strictly eliminated. Realistically, we may want to focus on a range of models that are qualitatively different in a single study. These should include models for which we have strong predictions (so as to put current opinion to the test) and models for which we have no strong predictions (to go beyond the state of the literature and advance theory). In fMRI studies, we can test our models in a variety of regions of interest or continuously throughout the measured volume using a searchlight approach [100]. Such more exploratory analyses represent another major source of important information. We can combine exploration and confirmation in a single study using multiple-testing correction and cross-validation.

Testing of a wide range of well-motivated models in multiple brain regions constitutes an approach that is simultaneously strongly hypothesis- and strongly data-driven 15, 101. This is the kind of approach we need to bridge the divide between modeling and experimental approaches, and to richly constrain brain theory with empirical data. We must resist two temptations: (i) to shy away from disconfirmation of prevailing bias and (ii) to restrict our analyses to be able to tell a better ‘story’. A story that crumbles upon consideration of a broader view of the available evidence (i.e., more comprehensive analyses of our own data) is clearly not worth telling.

### Engaging temporal dynamics

Representations are inherently dynamic, emerging over time as evidence from the sensory stream accumulates or recurrent computations converge on an interpretation of the input. At the level of higher cognition, thoughts emerge dynamically through the interplay of perception, long-term memory associations, and the current contents of working memory. Representational dynamics can be investigated by analyzing the representational geometry with a temporal sliding window. Recent studies have begun to move in this direction, using sliding-window decoding techniques for neuronal recordings [71] and human magnetoencephalography (MEG) 102, 103, 104 and electroencephalography (EEG) data [37].

### Improving characterizations of representational geometry

We might seek improvements to the approach of comparing representations by the rank correlation between their distance matrices. This approach does not require the (often questionable) assumption of a linear relationship between the distances, and deals gracefully with the fact that distances estimated from noisy pattern estimates are generally positively biased. However, it also discards potentially important geometric information. A promising complementary approach to comparing representations based on noisy data is pattern component modeling, in which the pattern variance is decomposed into components that correspond to experimental factors and noise [105].

Analysis of representational dissimilarity matrices is one important tool for understanding representational geometry. However, linear decoding remains useful as a straightforward test of linear separability (a particular feature of the representational geometry) and of the degree to which the linear decision boundary generalizes to different stimuli. Nonlinear decoders based on quadratic boundaries or radial basis functions [106] similarly reveal information that is available for immediate readout and might thus be considered explicit in the code, just like information available for linear readout. We will need a repertoire of specific models to test for a range of computationally relevant properties of the representational geometry.

### Considering different population codes and representational distance measures

Current studies have largely defined representations as spatial activity patterns (e.g., from fMRI or windowed spike counts), with the activity level in each voxel or neuron contributing one dimension to the space. However, population coding theory has explored a much wider range of possible codes, including temporal codes. For example, the representational dissimilarity of two stimuli could be measured by comparing the temporal order in which neurons fire their first spike in response to the stimulus [107] or relative to the Gamma cycle [108]. Another approach would be to use spatiotemporal or time–frequency patterns to define the representational space. In addition, a range of distance measures based on neuronal population spike trains deserves to be explored [109].

### Investigating plasticity and individual differences

One of the challenges in understanding the brain (or the mind) is that it is not a unitary object. Everyone is different, and our brains are continually plastic. If we are to understand the mind or the brain, we must be interested in interindividual variation and plasticity. Several studies suggest that the neural representation of visual stimuli is largely shared across people 19, 110, 111. Future work should elucidate how representational geometries are transformed through experience and whether, in addition to the component of the representation that is shared between individuals, there is a replicable individually unique component that can explain a person's unique perception and behavior.

## Concluding remarks

The study of representational geometry has provided novel insights into such diverse domains as vision and audition, categorical perception, memory encoding and recall, emotion, motor control, and higher cognitive processes including conscious awareness. Representational geometry can serve as a hub that enables us to connect experiment and theory at the crucial intermediate level of description, where computational network models meet massively multivariate data from electrophysiological population recordings and high-resolution functional imaging. Moreover, representational geometries can be compared between stages of processing, between model and brain representations, between individuals and species, and between brain and behavior. Many outstanding questions remain (Box 3). Future studies should systematically test wider ranges of models, including computational models, better integrate population-coding theory, reveal the representational dynamics, and elucidate representational plasticity and the individually unique component of representational geometries.

Box 3 **Outstanding questions**

• How are representational geometries transformed across stages of processing in vision and other modalities?
• How do perceptual representational geometries emerge over tens and hundreds of milliseconds as sensory information accumulates and recurrent computations converge?
• How do learning and expertise affect representational geometry?
• What aspects of behavior can we predict from the representational geometry at different stages of the perception-to-action cycle?
• Can representational geometries explain unique aspects of an individual's mind and behavior?
• Are impairments of perception and cognition in mental illness associated with atypical representational geometries? Can representational geometries be tracked over time to characterize the course of an illness and the effect of therapeutic interventions?
• What multivariate distance is best suited for measuring representational dissimilarities in neuronal or fMRI data?
• What mathematical concepts of topology and geometry might be useful for understanding neuronal population codes (e.g., non-Euclidean or nonmetric geometries)?
• How can neuronal coding theory best be brought to bear on analyses of representational geometry?
• The geometric model of similarity judgments and generalization behavior can help us understand neuronal population codes. Does the same hold true for feature-, analogy-, and transformation-based similarity models?
• What form of statistical inference best enables us to adjudicate between competing models that explain representational geometry?

## Glossary

Decoding model: model that takes brain activity patterns as input and predicts the experimental condition (e.g., the stimulus). Significant decodability indicates that information about the experimental condition is present in the region. A decoder can be used to model readout of a population code and predict the reflection of the code in downstream regions and behavior.
Encoding model: model that takes the stimulus as input and predicts brain activity. An encoder can be used to model brain processing of the stimulus, which gives rise to a population-code representation for a given area.
Explicit representation: neuronal representation of a stimulus property that allows immediate readout of the property by downstream neurons. If the property can be read out by means of a linear combination of the activities of the neurons or by a radial basis function, the property is explicitly represented. (For example, the category of a visual object is implicitly represented in the retina and explicitly represented in inferior temporal cortex.)
Geometric model of similarity: model of an internal multidimensional representational space originally devised to explain human dissimilarity judgments and patterns of generalization from examples to novel stimuli. By measuring brain activity patterns, we can attempt to find the neuronal implementation of the internal representational space. In the present context, this body of ideas is important because we can apply the concepts of similarity and generalization to gain a functional understanding of neuronal population codes and their transformation across stages of processing. The conceptual function of the geometric model for understanding neuronal computation is independent of its original goal, and its successes and limitations, as a cognitive theory of similarity judgments and generalization behavior.
Linear readout: decoding scheme based on a weighted sum of the activities of input neurons. Linear decoders are useful because they are realistic to fit given limited brain-activity data and any information they reveal about the stimulus can be thought of as explicitly represented (see above).
Multidimensional scaling: procedure by which we can arrange *n* items in a *d*-dimensional space such that their distances in the space best reflect their dissimilarities (by different metric or nonmetric criteria). The technique can be used, for example, to attempt to recover the internal representational space (which is typically assumed to be Euclidean) from dissimilarity judgments. It is also useful for producing two-dimensional arrangements that best visualize the distances of the items in a higher dimensional space, such as a neuronal population code. (Note that the MDS objective to best represent the original distances in *d* dimensions is distinct from the objective of principal components analysis to find the *d*-dimensional linear subspace that explains maximum variance.)
Pattern component modeling (PCM): analysis technique that decomposes the variance of brain activity patterns associated with a set of stimuli (or experimental conditions) into components that reflect different predefined factors such as the stimulus category, within-category variance, and measurement noise [105]. PCM can provide useful summary descriptions of the representational geometry.
Pattern-information analysis: approach to the analysis of brain activity data in which the activity patterns within a functional brain region are analyzed multivariately as a population code. For example, a cortical area may be recorded invasively with an array of electrodes or imaged by fMRI. The responses measured with electrodes or fMRI are typically also restricted to no more than a few hundred channels per brain region and can be viewed as a sample of the neuronal population activity. The subsampling means that a lot of information is lost in either case. Results should be interpreted as lower bounds for the information actually present in a region. fMRI is also compromised by the fact that voxels reflect neuronal activity only indirectly through hemodynamics. The response of a voxel to increased neuronal activity is thought to reflect a combination of subthreshold activity and neuronal firing, averaged locally across space and time.
Population code: scheme for encoding information thought to be important to the organism in the activity of a population of neurons. The simplest scheme uses only the firing rates of the neurons. More complex codes also use the information contained in the precise temporal patterns of spikes and their relationships between neurons. For each neuron, a tuning curve describes how the firing rate reflects particular stimulus properties. A population code for a particular type of information is ‘good’ when it represents the information in a format that can be read out by neurons receiving input from the population. Moreover, a good code should be efficient in the sense of achieving the required precision without wasting neuronal or energy resources.
Representational dissimilarity matrix (RDM): square matrix indexed horizontally and vertically by the stimuli (or experimental conditions) and containing a dissimilarity index in each cell, which compares the two brain-activity patterns associated with stimuli labeling the row and column. An RDM provides a useful characterization of the representational geometry (see below) for a limited set of experimental stimuli. If the same activity pattern estimates are used for the vertical and horizontal dimensions, the RDM is symmetric about a diagonal of zeros. If independent pattern estimates are used for the vertical and horizontal dimensions, the RDM contains entries comparing independent pattern estimates for identical stimuli (reflecting measurement noise) along its diagonal, and two alternative dissimilarity estimates for each stimulus pair in symmetric off-diagonal positions.
Representational geometry: geometry of a set of entities represented as points in a space spanned by the dimensions of a neuronal population code. Representational geometry focuses on the relationships between the entities, rather than on single entities, and on geometric properties including distances in the high-dimensional space rather than on differences in the activity of single neurons. Representational geometry provides a useful intermediate level of description that helps us abstract from idiosyncrasies of individual brains and highlights the representational properties that are key to the computational goals of the brain.
Represented information: mutual information between stimuli and response patterns. This comprises any differences between stimuli that can in principle be decoded from a neuronal population code. Given limited data, we cannot fit arbitrarily complex decoding models in practice. Thus, estimates of the represented information are negatively biased. The data processing inequality states that information can only be lost, never gained, along stages of processing. The retina has all the represented information about visual stimuli that any brain region has.
Similarity: subjective notion of the relationship between two objects that reflects the degree to which an organism distinguishes them. Similarity is inherently subjective, because any two non-identical objects will share some properties and differ in other properties. Judging similarity from object properties necessarily implies some choice and weighting of the properties. In the interest of its survival and reproduction, an organism should regard two objects as similar if they require the same behavioral response, for example, if one can replace the other (positive objects) or if both pose the same danger (negative objects). Which properties are relevant and irrelevant and how they should be weighted depend on the individual, its current goals, and the context. We can apply the concept of similarity not only at the level of the entire organism but also at the level of individual brain representations. This enables us to view neuronal computation as the stage-by-stage transformation of population-code representational similarity, whose function is to be understood in the larger context of the goals of brain information processing.
Single-neuron computational model: model that predicts the responses of a particular neuron to arbitrary stimuli. For example, responses of primary visual simple and complex cells can be modeled using localized linear filters of the input image and simple nonlinearities. For higher-level brain regions such as IT, it is currently impossible to fit single-neuron computational models. The problem is not only that the model space is insufficiently defined, but also that the number of parameters to be fitted (for any plausible model space) is too large given the number of stimuli for which response data can be acquired.
